# Cost and cost-effectiveness of indoor residual spraying with pirimiphos-methyl in a high malaria transmission district of Mozambique with high access to standard insecticide-treated nets

**DOI:** 10.1186/s12936-021-03687-1

**Published:** 2021-03-10

**Authors:** Sergi Alonso, Carlos J. Chaccour, Joseph Wagman, Baltazar Candrinho, Rodaly Muthoni, Abuchahama Saifodine, Francisco Saute, Molly Robertson, Rose Zulliger

**Affiliations:** 1 Wellcome Centre for Integrative Parasitology, Institute of Biodiversity, Animal Health and Comparative Medicine, University of Glasgow, Glasgow, G12 8QQ UK; 2 Centro de Investigação Em Saúde de Manhiça, Maputo, Mozambique; 3 ISGlobal, Hospital Clínic - Universitat de Barcelona, Barcelona, Spain; 4 PATH, Washington, DC USA; 5 Programa Nacional de Controlo da Malaria (NMCP), Maputo, Mozambique; 6 Abt Associates, PMI-AIRS Mozambique, Maputo, Mozambique; 7 U.S. President’s Malaria Initiative, US Agency for International Development, Maputo, Mozambique; 8 U.S. President’s Malaria Initiative and Malaria Branch, Division of Parasitic Diseases and Malaria, U.S. Centers for Disease Control and Prevention, Maputo, Mozambique

**Keywords:** Vector control, Indoor residual spraying, Insecticide-treated net, Economic evaluation, Mozambique

## Abstract

**Background:**

As malaria cases increase in some of the highest burden countries, more strategic deployment of new and proven interventions must be evaluated to meet global malaria reduction goals.

**Methods:**

The cost and cost-effectiveness of indoor residual spraying (IRS) with pirimiphos-methyl (Actellic®300 CS) were assessed in a high transmission district (Mopeia) with high access to pyrethroid insecticide-treated nets (ITNs), compared to ITNs alone. The major mosquito vectors in the area were susceptible to primiphos-methyl, but resistant to pyrethoids. A decision analysis approach was followed to conduct deterministic and probabilistic sensitivity analyses in a theoretical cohort of 10,000 children under five years of age (U5) and 10,000 individuals of all ages, separately. Model parameters and distributions were based on prospectively collected cost and epidemiological data from a cluster-randomized control trial and a literature review. The primary analysis used health facility-malaria incidence, while community cohort incidence and cross-sectional prevalence rates were used in sensitivity analyses. Lifetime costs, malaria cases, deaths and disability-adjusted life-years (DALYs) were calculated to determine the incremental costs per DALY averted through IRS.

**Results:**

The average IRS cost per person protected was US$8.26 and 51% of the cost was insecticide. IRS averted 46,609 (95% CI 46,570–46,646) uncomplicated and 242 (95% CI 241–243) severe lifetime cases in a theoretical children U5 cohort, yielding an incremental cost-effectiveness ratio (ICER) of US$400 (95% CI 399–402) per DALY averted. In the all-age cohort, the ICER was higher: US$1,860 (95% CI 1,852–1,868) per DALY averted. Deterministic and probabilistic results were consistent. When adding the community protective effect of IRS, the cost per person protected decreased (US$7.06) and IRS was highly cost-effective in children U5 (ICER = US$312) and cost-effective in individuals of all ages (ICER = US$1,431), compared to ITNs alone.

**Conclusion:**

This study provides robust evidence that IRS with pirimiphos-methyl can be cost-effective in high transmission regions with high pyrethroid ITN coverage where the major vector is susceptible to pirimiphos-methyl but resistant to pyrethroids. The finding that insecticide cost is the main driver of IRS costs highlights the need to reduce the insecticide price without jeopardizing effectiveness.

**Trial registration**: ClinicalTrials.gov identifier NCT02910934 (Registered 22 September 2016). https://clinicaltrials.gov/ct2/show/NCT02910934?term=NCT02910934&draw=2&rank=1

**Supplementary Information:**

The online version contains supplementary material available at 10.1186/s12936-021-03687-1.

## Background

Effective vector control programmes have direct health benefits by reducing vector-borne diseases burden and have increased labour productivity, economic development; and strengthened health systems [1]. Worldwide reductions in malaria morbidity and mortality are largely due to the scale-up of vector control strategies for malaria prevention, mainly insecticide-treated nets (ITNs) and indoor residual spraying (IRS), as well as the introduction of effective therapies and reliable diagnostic tools [2]. As such, the continued reduction of the global malaria burden is successfully shrinking the geographical distribution of the disease [3] and has laid the foundation for recent malaria elimination initiatives [4].

Despite these gains, ten sub-Saharan African countries with the highest malaria burden, accounting for more than half of the global disease burden, registered an increase in the number of malaria cases in 2017. [5]. This increase jeopardizes the achievement of current malaria burden targets [6]. This trend reversal is in part explained by reduced malaria prevention coverage, reduced effectiveness of pyrethroid insecticides used on ITNs due to increasing pyrethroid resistance among the primary malaria vectors, lack of political commitment, inadequate guidance to adapt new strategies in local settings of high burden countries, stagnation of investments and insufficient funds for malaria control and limited national cooperation across-sectors [7].

Optimizing the use of vector control interventions is one of the key tools to meeting global targets [6]. The effectiveness and cost-effectiveness of malaria vector control strategies are well documented [8, 9], and currently most sub-Saharan African countries recommend the implementation of either ITNs or IRS as preventive measures in malaria risk regions, in alignment with World Health Organization (WHO) recommendations [5]. A key factor for the success of both interventions is the effectiveness of the insecticides applied. Despite their individual efficacy, few studies have evaluated the joint effect of both interventions [10–12], particularly in high transmission settings. Protopopoff et al. [13], found no additional benefit of combining ITN and IRS when effective insecticides were used for both interventions, but evidence on the combined effectiveness is still inconclusive [14]. The WHO Guidelines for Malaria Vector Control recently declared the need to close this evidence gap with reliable economic and epidemiological data to determine the cost-effectiveness of different packages of vector control interventions to inform policy makers [1].

With around 10 million cases every year, representing 5% of all malaria cases worldwide [5], Mozambique has one of the highest malaria burdens. This burden is heterogeneously distributed; while most parts of the central and northern provinces remain high transmission areas with prevalence of 70% and above in children under five years of age (U5), some districts in the southern provinces have lower incidence and the prevalence is below 1% in children U5 [15]. Approximately 66% of the population in Mozambique has access to an ITN and 11% of households are covered by IRS [5]. A limited number of households benefit from the protective effect of both interventions simultaneously as the country has implemented nationwide ITN campaigns and targeted IRS in high burden and pre-elimination areas. There is, however, insufficient data to inform the appropriateness and necessary scale of this combination in high-burden areas of the country.

Furthermore, emerging resistance to insecticides amongst mosquitoes might threaten vector control strategies. The Mozambican Ministry of Health has confirmed vector resistance to pyrethroids, organochlorines, such as dichlorodiphenyltrichloroethane (DDT), and carbamates, but vectors remain susceptible to organophosphates [5]. In some districts of central Mozambique the main vector species, *Anopheles gambiae* and *Anopheles funestus*, had documented resistance to pyrethroids as early as 2010 [16–19]. In this context, information on the effectiveness and cost-effectiveness of using new insecticides for vector control strategies is critical.

In order to address this evidence gap, a cluster-randomized controlled trial was conducted from 2016 to 2018 to assess the effectiveness of IRS with micro-encapsulated pirimiphos-methyl (Actellic®300 CS) in addition to pyrethroid ITNs in the high-transmission district of Mopeia, central Mozambique, as compared to pyrethroid ITNs alone [20]. The primary vectors in the areas are susceptible to pirimiphos-methyl and resistant to pyrethroids. The present study assessed the economic cost of the 2016 and 2017 IRS campaigns, as well as the cost-effectiveness of the combined effect of IRS and pyrethroid ITNs in Mopeia, as compared to pyrethroid ITNs alone.

## Methods

### Study design and description of interventions

The study was conducted from November 2016-October 2018 in Mopeia, central Mozambique, a rural district with poor socio-economic indicators and in a high malaria burden province: 68% prevalence in children U5 in 2015 [15, 21]. The population of the district in 2018 was 136,520 inhabitants. *Anopheles funestus sensu lato* (*s.l*.) and, to a lesser extent *An. funestus sensu stricto* (*s.s.*) and *An. gambiae s.l*., are the dominant local vectors. At baseline in 2017 *An. gambiae s.l.* was susceptible to pyrethroids and pirimiphos-methyl, but by 2018 there was emerging resistance in this vector to both alphacypermethrin and deltamethrin. There are no 2017 *An. funestus s.l.* data available, but *An. funestus s.l.* tested from Mopeia in 2018 were susceptible to DDT and pirimiphos-methyl but resistant to alphacypermethrin, deltamethrin and bendiocarb [22]. IRS with a microencapsulated formulation of the organophosphate insecticide pirimiphos-methyl was implemented in 43 IRS-assigned clusters in October–November 2016 and 2017 to provide protection during the 2017 and 2018 high transmission seasons (December to April). The control group consisted of 43 clusters that did not receive IRS. The entire district, including the 86 study clusters [20], was covered by routine and campaign distribution of pyrethroid ITNs, receiving over one ITN per inhabitant in 2013 with baseline ownership across the district of 61–63% in 2016. Most of these ITNs at baseline had been distributed through antenatal care services and through a mass campaign in 2013. An additional 120,765 pyrethroid ITNs were distributed through a mass campaign in June-July 2017 during study data collection, raising ownership to 90%. Additional details of the intervention, trial characteristics and data collection can be found elsewhere [20].

The study received approved from the Institutional Review Board of the CISM (CIBS-CISM), the National Bioethical Committee (Comité Nacional de bioética para a Saúde de Moçambique, CNBS), and PATH’s Research Ethics Committee (REC) and was considered to be human subjects research with non-engagement by US Centers for Disease Control and Prevention (CDC) and approved by the US President’s Malaria Initiative (PMI) Operational Research Committee.

### Decision analysis

A combination of decision analysis and life table analysis were used to evaluate the cost-effectiveness of the combined effect of IRS and ITNs as compared to pyrethroid ITNs alone on (1) a theoretical cohort of 10,000 children U5 and, (2) a theoretical cohort of 10,000 individuals of all ages, followed over their lifetime, based on the Mozambican average life expectancy [10, 23]. For each cohort, lifetime costs and health effects associated to each study arm were compared. Each year each cohort was at risk of malaria according to the corresponding malaria infection incidence [22], and mortality rate [24], and individuals were taken out of the model based on a non malaria-related mortality rate [25]. Table 1 shows the input parameters used in the model, which were based on primary epidemiological impact data from the trial. Mortality and disability weight parameters were based on values and distributions obtained from peer-reviewed literature.

**Table 1:** Input variables and associated confidence intervals and ranges used in the deterministic and probabilistic sensitivity analysis for the cost-effectiveness evaluation of vector control strategies in Mopeia, Mozambique

Inputs	Likeliest	(Low–high)	Probability distribution	Source
Costs estimates (US$)				
IRS cost per person protected (Min–Max)	8.26	(7.25–9.27)	Gamma	Project data
Health system costs (IQR)				
HS—uncomplicated malaria	4.34	(4.32–4.35)	Gamma	[26]
HS—severe malaria	36.97	(18.03–44.09)	Gamma	[26]
Household costs (all ages) (IQR)				
Uncomplicated malaria—HH direct cost	0.74	(0.05–0.19)	Gamma	[26]
Uncomplicated malaria—HH indirect cost	16.67	(0.00–22.38)	Gamma	[26]
Severe malaria—HH direct cost	3.54	(0.60–6.31)	Gamma	[26]
Severe malaria—HH indirect cost	154.30	(31.45–87.71)	Gamma	[26]
Household costs (children U5) (IQR)				
Uncomplicated malaria—HH direct cost	0.92	(0.00–1.05)	Gamma	[26]
Uncomplicated malaria—HH indirect cost	7.23	(0.00–5.90)	Gamma	[26]
Severe malaria—HH direct cost	4.68	(1.99–6.31)	Gamma	[26]
Severe malaria—HH indirect cost	61.23	(47.17–71.60)	Gamma	[26]
DALYs				
Discount rate (range)	0.03	(0.00–0.05)	Point estimate	Assumption
Average age (all ages)	15	-	Point estimate	[21]
Average age (children U5)	1	-	Point estimate	[22]
Life expectancy at birth (95% CI)	57.07	(49.73–64.61)	Lognormal	[23]
Length of disability: uncomplicated malaria (range)	0.01096	(0.00548–0.01644)	Gamma	Assumption
Length of disability: severe malaria (range)	0.02739	(0.02054–0.03424)	Gamma	Assumption
Disability weight: uncomplicated malaria (range)	0.051	(0.032–0.074)	Lognormal	[27]
Disability weight: severe malaria (range)	0.133	(0.088–0.190)	Lognormal	[27]
Effects measurement				
% malaria cases severe (range)	0.00517	(0.00259–0.00776)	Beta	[22]
Malaria infection incidence (95% CI)				
Incidence rate ratio—health facility-based (all ages)	0.78	(0.77–0.79)	Lognormal	[22]
Incidence rate ratio—health facility-based (children U5)	0.76	(0.75–0.78)	Lognormal	[22]
Incidence rate ratio—community-based (children U5)	0.82	(0.79–0.86)	Lognormal	[22]
Malaria prevalence in 2018 (95% CI)				
Odds ratio (all ages)	0.70	(0.49–1.00)	Lognormal	[22]
Odds ratio (children U5)	0.54	(0.31–0.92)	Lognormal	[22]
Mortality estimates (per 10,000 individuals) (range)				
Case fatality rate malaria (all ages)	0.000873	(0.000655–0.001091)	Beta	[24]
Case fatality rate malaria (children under five)	0.005205	(0.003904–0.006506)	Beta	[24]
Non-malaria mortality rate	0.0116	(0.0058–0.0174)	Beta	[25]
Others (US$)				
Threshold cost-effectiveness (3 times 2018 GDPpc)	1470.60	-	Point estimate	[35]

*Children U5* children under five years of age, *CI* confidence interval, *DALY* disability-adjusted life-year, *GDPpc* gross domestic product per capita, *HH *household, *HS* health system, *IQR* inter-quartile range, *IRS* indoor residual spraying

Annual frequency of malaria infection was estimated to result in an uncomplicated or severe episode according to the probability of disease severity extracted from the trial. Household and health system costs of malaria care for each uncomplicated and severe malaria case in this setting have been previously described [26] and are summarized below under economic burden of malaria. The median age at study baseline (one year) was used for the theoretical cohort of children U5, and the most recent projected median age of the district population (15 years) was used for the theoretical all age cohort [21]. Migration from study site, malaria sequelae and other disease manifestations associated with malaria, such as anaemia, were disregarded due to lack of reliable data available in the district.

### Health effects

Epidemiological data from the trial were used to assess the health effects of the IRS intervention (Table 1) [22]: IRS with pirimiphos-methyl in an area with high standard ITN coverage reduced malaria incidence measured at health facility by 22% in individuals of all ages, and by 24% in children U5. At community-level the reduction was lower: 18% in children U5. We used the malaria infection incidence associated with each study arm measured by monthly passive case surveillance records from health facilities and community health workers to calculate the corresponding number of malaria cases and deaths for the two theoretical cohorts (10,000 children U5 and 10,000 individuals of all ages), differentiating between uncomplicated and severe malaria cases [22]. Due to the absence of reliable mortality data from the trial, we used malaria and non malaria-related mortality rates reported for the country [24, 25]. Disability-adjusted life-years (DALYs) were further calculated for each study arm and target population. DALYs accounted for the accrual of malaria-related years of life lost and years lost due to disability for uncomplicated and severe malaria. DALYs were calculated using unweighted age-specific life expectancy [23], moderate and severe malaria-related disability weights reported by the Global Burden of Disease [27], and a 3% discount rate in accordance with WHO guidance. The primary health outcomes were the number of DALYs averted for each theoretical cohort, while the secondary outcome measures were the number of malaria-related cases and deaths averted for the theoretical cohorts considered. Alternative epidemiological data from the trial’s other methods were used for the sensitivity analyses: (1) malaria infection incidence from an active case surveillance at community level, and (2) malaria prevalence from the 2018 cross-sectional study [22]. Table 2 summarizes the malaria-related measures used in the current study.

**Table 2:** Definition of malaria-related epidemiological measures used in the model

Health facility-based malaria incidence	Calculated from confirmed malaria cases seeking care at public health facilities and community health workers in Mopeia, measured following an enhanced passive surveillance approach *Used in the main analysis for both cohorts*
Community-based malaria incidence	Calculated from an active cohort of children under five years of age at community level *Used in the univariate sensitivity analysis for the children under five cohort*
Malaria prevalence	Calculated in individuals of all ages for both study arms from the 2018 cross-sectional survey at the peak of the transmission season (April – May) *Used in the univariate sensitivity analysis for both cohorts*
Severe malaria case	Malaria case requiring hospital admission. Computed from the total number of malaria infected according to the malaria severity ratio obtained from the trial *Used in the main analysis for both cohorts*
Uncomplicated malaria case	Malaria case not requiring hospital admission
Malaria case fatality rate	Malaria-associated case fatality rate estimated by the Global Burden of Disease (GBD) for the most current estimate (2017). Different estimates were used for the children under five and all age cohort *Used in the main analysis for both cohorts*
IRS with community-wide protective effect	IRS achieving high coverage (> 85%) so that the population target benefits from a “mass effect” on the vector population [33] *Used in the univariate sensitivity analysis for both cohorts*

*IRS* indoor residual spraying

### Interventions costs

Abt Associates provided resources and costs incurred for IRS activities in Mopeia, which were prospectively collected for the two spray campaigns. Following an ingredient-based approach, all resources associated with spraying activities were classified and estimated [28]. Resources considered in the analysis included the personnel employed for spraying and monitoring activities in Mopeia, vehicle leasing and fuel, equipment, training and communication activities, commodities, the use of buildings, microplanning of activities, as well as salaries of personnel in Maputo and the US headquarters attributable to Mopeia. IRS was implemented in the study district and in six additional routine implementation districts in Zambézia province by the President’s Malaria Initiative Africa IRS project (PMI AIRS) and the Mozambican Ministry of Health with all financial costs paid for by PMI. Personnel, project management and monitoring and evaluation costs from headquarters in the United States, Mozambique and Zambézia central offices were allocated according to time devoted to Mopeia. The percentage of time spent in Mopeia for each year was calculated as the total number of structures sprayed in Mopeia divided by the total number of structures sprayed in all districts supported by PMI AIRS.

Insecticide was procured by the Government of Mozambique Global Fund grant with a co-payment by UNITAID through the NgenIRS partnership. The full insecticide economic cost paid by both the Global Fund and UNITAID was included in this analysis.

Spraying activities in Mopeia were coordinated from three operational bases within the district. There was a district-level warehouse and storekeeper, and a supervisor and a storekeeper in each base. Spray teams included a team leader and five spray operators. Brigade supervisors then coordinated and supervised brigades comprised of three teams. In addition to Ministry of Health staff who received per diems for their time working on the IRS campaign, Mopeia had one full-time PMI AIRS district coordinator. Salaries for the Ministry of Health staff were prorated based on the full-time equivalents allocated to the IRS campaigns. All other staffing related to the campaign were temporary hires: four storekeepers, three site supervisors, four brigade supervisors, 12 team leaders and 60 spray operators. Additionally, 12 community mobilizers, two pump technicians, eight washers and five security guards supported the activities. Insecticide and other commodities were sent from the provincial capital to the district storehouse. In the district, two vehicles covered the transport to operational sites and were also used to transport teams for spraying activities.

In addition to micro-costing of all materials and staffing related to implementation of the IRS campaign, estimates also included the use of resources already in place that did not necessarily imply a direct payment from IRS, such as the district office rental; thus capturing the economic costs of the programme [29]. The costs for all items procured in Mozambique were recorded in Mozambican meticais and later converted to United States dollars (US$) according to the official exchange rate [30]: 63.06 meticais/US$ in 2016 and 63.58 meticais/US$ in 2017. Quantities were inflated-adjusted [31] using the 2016 GDP deflator (13.68%), and annualization of capital costs were subject to a standard discount rate of 3% [29, 32]. IRS costs were expressed as cost per (1) structure sprayed and, (2) person protected. We further computed the IRS cost per person protected adding the community-wide protective effect, which considered the whole target population, due to the high coverage achieved (> 85%) [33]. Purchase and distribution costs of ITNs were not included in this analysis, as ITN ownership was not statistically different between study arms [22].

### Economic burden of malaria

Households and health system costs associated with a malaria case have been previously described [26]. Households care-seeking costs included formal and informal care. Following a societal perspective, the costs of an uncomplicated and severe malaria case of each study arm were calculated as the lifetime summation of the health system and household costs associated with malaria. Household costs were reported including (1) only household direct costs and, (2) direct and indirect household costs, which considered the time lost due to illness. Costs were expressed in 2018 US$ and discounted at a standard 3% rate to account for time preference.

### Statistical analyses

The expected lifetime costs and malaria-related DALYs were calculated for each cohort, alongside the lifetime morbidity and mortality events associated with receipt of IRS and no IRS. Incremental cost-effectiveness ratios (ICERs) were calculated as the lifetime incremental costs divided by DALYs averted due to the IRS intervention in a context of high pyrethroid ITN coverage. Incremental costs were calculated as the summation of lifetime IRS costs in the IRS arm minus the lifetime cost savings due to a reduction of malaria burden between study arms. ICERs were calculated considering (1) household direct and indirect costs and, (2) only household direct costs. In accordance with recommendations from the WHO [34], the maximum level for which combining IRS and ITNs was considered cost-effective compared to ITNs alone was US$1,470.60 per DALY averted; three times the 2018 Mozambican gross domestic product (GDP) per capita [35]. The highly cost-effective threshold was US$490.20 per DALY averted (GDP per capita).

A deterministic cost-effectiveness analysis was conducted using the most likely values of the parameters in the model. Uncertainty was incorporated by conducting a sensitivity analysis in which we evaluated univariate changes on ICERs due to changing the lower and higher values of key parameters (Table 3). Changes considered for sensitivity analyses in both cohorts included: (1) using the malaria prevalence obtained in 2018 from the cross-sectional survey instead of using data on malaria incidence from passive surveillance [22], (2) increasing and decreasing malaria incidence by 10% in individuals of all ages; (3) reducing the IRS cost per person protected to US$7.06 [36], due to adding the community-wide protective effect of achieving high IRS coverage [33], and increasing the IRS cost by 50%; (4) decreasing the malaria-related mortality rate in individuals of all ages to 6.134/10,000 [24], and increasing it to 7.294/10,000 (10% increase); (5) reducing the percentage of severe malaria cases by 20% and increasing it to a severity rate of 0.0122 [37]; (6) modifying the discount rate (0% and 5%) and; (7) modifying the health system costs by assuming no cost of screening fever (US$3.54) and applying higher malaria commodity costs (US$5.12) [26]. Additionally, changes for the children U5 cohort included: (1) reducing the IRS-arm incidence by 30%, and using the incidence obtained at community level by an active case surveillance (incidence rate ratio = 0.82) [22]; and (2) increasing and reducing malaria-related mortality rate in 20% in children U5. Tornado diagrams were used to represent these changes on the ICERs due to univariate sensitivity analyses.

**Table 3:** Univariate sensitivity analysis: changes in selected parameters to account for uncertainty

Parameter	Lower value	Upper value	Reference
Parameters changed for the cohort of children under five years of age (U5)			
IRS-arm malaria incidence: children U5	30% reduction	IRR = 0.82	Assumption, [22]
Malaria mortality rate (children U5)	20% reduction	20% increase	Assumption
Parameters changed for both cohorts (children U5 and individuals of all ages)			
Malaria prevalence (instead of incidence)	ORall = 0.70 and ORchildrenU5 = 0.54		[22]
IRS-arm malaria incidence: all ages	10% reduction	10% increase	Assumption
IRS cost per person protected	US$7.06	50% increase	[36], assumption
Malaria mortality rate (all ages)	6.134/10,000	10% increase	[24], assumption
% severe malaria over all malaria cases	20% reduction	0.0122	Assumption, [37]
Discount rate	0%	5%	Assumption
Health system cost—uncomplicated case	US$3.54	US$5.12	[26]

Changes on the average health system costs associated with malaria are based on: (1) assuming no cost of screening fever (US$3.53) and higher commodity costs (US$5.12). Malaria prevalence rates from cross-sectional studies were used as alternative to health facility-based malaria incidence rates in the main analysis

*IRR* malaria incidence risk ratio, *IRS*  indoor residual spraying, *OR* odd ratio (malaria prevalence rates calculated from both study arms)

A probabilistic approach was further adopted in which the model parameters followed probability distributions (Table 1). Parameters’ distributions were extracted from trial primary data or, in their absence, were constructed assuming between 25–50% increase/decrease in the range of the parameters’ most likely values. Monte Carlo simulations (10,000 iterations) were used to produce a point estimate and 95% CIs for the incremental costs, DALYs averted and ICERs. Incremental costs and DALYs averted were graphically represented in two-dimensional cost-effective planes. Acceptability curves were employed to represent the probability of the IRS arm to be cost-effective depending on a theoretical willingness-to-pay per DALY averted. MS Excel 2016 (Visual Basic for Applications) and Stata v15 (StataCorp) were used for data analysis.

## Results

### Indoor residual spraying (IRS) campaigns

The IRS campaigns, which covered half of the district of Mopeia, lasted 42 days in 2016 and 50 days in 2017 and sprayed a similar number of structures each year, almost 17,000 sprayable structures (Additional file 1: Table S1). Most of the district families lived in a household made up by a single structure (75%), some lived in a space with two structures (18%), and the remaining lived in a household with three or more structures (7%). The most common materials used in wall construction were clay blocks (40%), wood (35%) and bricks (11%). The campaigns reached approximately 85% of the sprayable structures in the IRS arm.

The average total cost of IRS using pirimiphos-methyl in the district was US$607,122, and the 2017 campaign was more expensive (Table 4). Insecticide costs represented approximately half the total costs (51%). Other costs included project management (17%), personnel (6%), vehicles (10%), equipment (6%), monitoring and evaluation (3%), training (3%), other commodities (3%) and buildings (2%). The programme average cost per structure sprayed was US$36.28 per year, yielding an average cost per person protected of US$8.26 per year. The latter reduced to US$7.06 per person protected when considering the community-wide protective effect due to the high IRS coverage achieved [33].

**Table 4:** Inflation-adjusted costs of indoor residual spraying activities in the district of Mopeia in 2016 and 2017 (US$)

	Average economic cost of IRS campaigns		2016 campaign		2017 campaign	
		% of total	Economic cost	% of total	Economic cost	% of total
Personnel	35,019.67	5.77%	32,275.28	5.81%	37,764.06	5.74%
Vehicles	60,299.66	9.93%	54,894.23	9.87%	65,705.10	9.98%
Equipment	39,048.14	6.43%	40,273.10	7.24%	37,823.19	5.75%
Training	16,595.22	2.73%	16,978.43	3.05%	16,212.02	2.46%
Insecticide	312,006.37	51.39%	263,430.58	47.38%	360,582.16	54.78%
Commodities	15,160.09	2.50%	17,295.04	3.11%	13,025.14	1.98%
Buildings	9,745.97	1.61%	8,945.52	1.61%	10,546.43	1.60%
Project management	100,453.00	16.55%	104,803.45	18.85%	96,102.55	14.60%
Monitoring and evaluation	18,793.48	3.10%	17,088.59	3.07%	20,498.37	3.11%
Total	607,121.61		555,984.21	100%	658,259.00	100%
Cost per structure sprayed	36.28		33.70		38.87	
Cost per person protected	8.26		7.25		9.27	
Cost per person protected with community-wide effect^1^	7.06		6.23		7.89	

^1^ The value was computed as the total IRS cost divided by the total target population, as it was assumed that a community-wide effect would protect individuals of non-sprayed households due to the high coverage achieved in the campaigns (85%)

**Table 5:** Probabilistic cost-effectiveness results of the combined effect of indoor residual spraying (IRS) and insecticide-treated nets (ITNs) on a theoretical cohort of 10,000 (A) children under five years of age and (B) individuals of all ages

	Theoretical cohort of children U5			Theoretical cohort of individuals of all ages		
	Point estimate	95% CI lower	95% CI upper	Point estimate	95% CI lower	95% CI upper
Incremental costs = intervention costs—malaria cost savings						
Direct costs only	1,633,402.91	1,630,687.28	1,636,118.53	1,578,361.96	1,575,844.28	1,580,879.64
Direct and indirect costs	1,128,662.46	1,125,185.47	1,132,139.45	1,138,624.72	1,135,000.29	1,142,249.14
Incremental effects						
Total cases averted	46,851	46,811	46,889	33,909	33,880	33,936
Uncomplicated cases averted	46,609	46,570	46,646	33,734	33,706	33,760
Severe cases averted	242	241	243	175	174	176
Deaths averted	94	93	94	30	29	30
DALYs averted	2,856.76	2,850.34	2,863.18	624.39	622.66	626.12
ICERs: incremental cost per DALY averted						
ICER—direct costs only	579.46*	577.86*	581.05*	2,578.68	2,570.55	2,586.80
ICER—direct and indirect costs	400.32**	398.82**	401.82**	1,860.08	1,852.17	1,867.98

Results from the Monte Carlo simulations with 10,000 iterations using the parameter values and distributions specified in Table 1

*Children U5* children under five years of age, *CI*  confidence interval, *DALY*  disability-adjusted life-year, *ICER* incremental cost-effectiveness ratio

*Implies that the combination of IRS with pirimiphos-methyl and pyrethroid itns is cost-effective compared to pyrethroid itns alone, according to the WHO-Choice threshold of three times the GDP per capita

**Implies the strategy is highly cost-effective

### Cost-effectiveness analyses

**Fig. 1:**
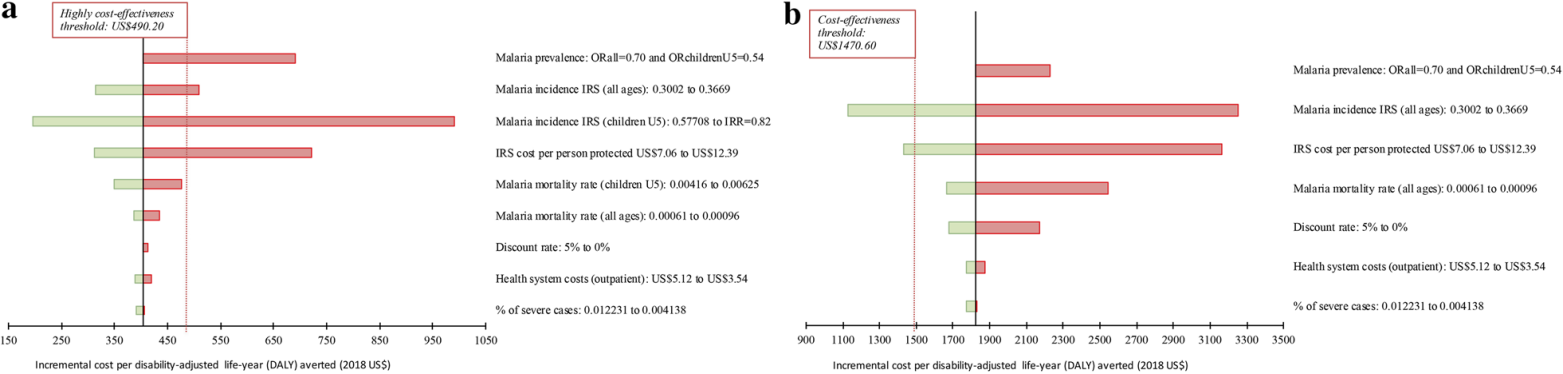
Tornado diagrams showing results from univariate sensitivity analyses. Changes on the deterministic value of the incremental cost-effectiveness ratio (ICER) by changing the value of selected parameters when considering households’ direct and indirect costs (US$). Analyses consider: **a** children under five years of age (pre-adjustment ICER of US$403.92) and **b** individuals of all ages (pre-adjustment ICER of US$1,821.86). Changes on the selected parameters had no impact on ICERs in children under five years of age or individuals of all ages with the exception of changes on the efficacy of IRS with pirimiphos-methyl and the insecticide price, which made the intervention cost-effective in individuals of all ages. The vertical lines represent the maximum cost-effectiveness thresholds. *DALY* disability-adjusted life-year, *IRS* indoor residual spraying, *OR* odd ratio

Using passive case surveillance and considering households’ direct and indirect costs, a deterministic cost-effectiveness analysis demonstrated that the combination of IRS with pirimiphos-methyl and pyrethroid ITNs was highly cost-effective compared to ITNs alone in the children U5 cohort (ICER = US$404) but not for the cohort of individuals of all ages (ICER = US$1,822) (Additional file 2: Table S2). The tornado diagrams (Fig. 1) confirmed that, amongst all parameters, the malaria infection incidence and the IRS cost per person protected had the higher impact on ICERs. For the all-ages cohort, both (1) the decrease in the IRS cost per person protected to US$7.06, which incorporated the community-wide protective effect, or (2) a 15% reduction in the IRS-arm incidence rate, made the intervention cost-effective, compared to the use of standard ITNs alone. With these changes in the children U5 cohort, the intervention remained highly cost-effective. Univariate changes of the remaining key parameters did not result in changes in the cost-effectiveness interpretation.

Probabilistic results, presented in Table 5, were consistent with those obtained in the deterministic analysis. For the children U5 cohort, the expected lifetime incremental costs were US$1,128,663 (95% CI 1,125,186–1,132,140), which included indirect household costs, averting 46,609 (95% CI 46,570–46,646) uncomplicated cases, 242 (95% CI 241–243) severe cases and 94 (95% CI 93–94) deaths associated with malaria. The incremental costs were US$400 (95% CI 399–402) per DALYs averted and US$580 (95% CI 578–581) per DALY averted when disregarding indirect household costs. Conversely, the intervention was not cost-effective for the cohort of all ages: US$1,860 (95% CI 1,852–1,868) per DALY averted and US$2,579 (95% CI 2,571–2,567) per DALY averted without indirect household costs. However, the combined effect of IRS and ITNs was able to avert 33,734 (95% CI 33,706–33,760) uncomplicated cases, 175 (95% CI 174–176) severe cases and 30 (95% CI 29–30) malaria deaths during the lifespan of the cohort.

**Fig. 2:**
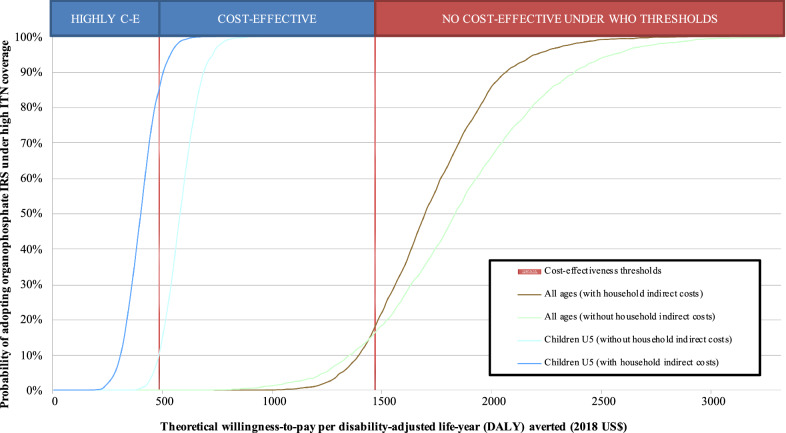
Cost-effectiveness acceptability curves for the cohort of children under five years of age and individuals of all ages. The curves graphically represent the probability of adopting IRS with pirimiphos-methyl under high pyrethroid insecticide-treated nets (ITNs) being cost-effective at specific willingness-to-pay values, compared to not adopting IRS with pirimiphos-methyl under high pyrethroid ITN coverage. The vertical lines correspond to the standard cost-effectiveness thresholds by the World Health Organization. *CE* cost-effectiveness, *Children U5* children under five years of age, *DALY* disability-adjusted life-year, *IRS* indoor-residual spraying

A graphical representation of the incremental costs and DALYs averted can be found in Additional file 3: Fig. S1. Most of the iterations were concentrated below the threshold line in the cohort of children U5 and above the line in the cohort of individuals of all ages, denoting the probabilistic analysis was cost-effective only for the cohort of children U5. Considering households’ indirect costs, for a theoretical willingness-to-pay of US$1,470.60, the combination of IRS and ITNs was cost-effective compared to ITNs alone for 100% of the simulations in children U5, and 18% of them in individuals of all ages (Fig. 2). In children U5 the intervention was highly cost-effective in 85% of the simulations for a theoretical willingness-to-pay of US$490.20.

## Discussion

These findings demonstrate that the combination of IRS with pirimiphos-methyl and ITNs with an insecticide to which vectors are resistant [38], can be highly cost-effective in high transmission settings amongst children U5, but not cost-effective amongst the all age population, as compared to ITNs alone. Sensitivity analyses, including using data from different study methods [22], confirmed that these results were relatively stable, but that reductions in IRS cost or increases in campaign effectiveness would influence the cost-effectiveness determination for both populations. IRS was highly cost-effective amongst children U5 when calculated using data from the passive surveillance and remained cost-effective when using effectiveness data from the active case surveillance and the 2018 cross-sectional study. Importantly, the ICER reduced to US$312 per DALY averted when adding the community-wide protective effect of IRS (IRS cost per person protected of US$7.06) (Table 4). Conversely, the adoption of IRS with pirimiphos-methyl was not cost-effective in individuals of all ages as the lower protective effect shown in the all-age impact data could not overcome the high IRS cost per person protected in the district. However, adding the community-wide protective effect of achieving high coverage would make the adoption of IRS with pirimiphos-methyl cost-effective in the high transmission district of Mopeia (ICER = US$1,431).

This study found that the yearly organophosphate IRS campaigns in Mopeia achieved high coverage rates at a high cost (US$607,122), mostly due to the high insecticide price, responsible for 51% of the total cost and the existence of relatively high overheads and allowances to allocate to the district. Hence, the average IRS cost per person protected in Mopeia (US$8.26) was amongst the highest compared with other non-adjusted estimates reported in the literature (Additional file 4: Table S3) [9, 10, 29, 39–46]. However, this is one of the first studies reporting IRS cost of new insecticide products, which are considerably more expensive than products that have been phased out for environmental reasons or due to resistance, such as DDT and pyrethroids [47]. The IRS cost per person protected obtained in this study differed from the ones reported by PMI in Mozambique: US$6.68 in 2016 and US$5.73 in 2017 [36]. Such differences can be explained by the total population covered. Despite the high coverage achieved, the denominator (~ 71,000 individuals) was lower than the one obtained for the seven districts sprayed in the province (1.7 million individuals); raising, thus, the marginal cost to protect one individual in Mopeia. Increasing economies of scale emerge when IRS campaigns expand to larger administrative areas [9, 36], or are directly implemented by the Ministry of Health without more expensive partner involvement. Nevertheless, the finding that the IRS cost would need to be reduced to meet the cost-effectiveness threshold in the all-age population and that this is largely driven by the insecticide cost underscores the importance of initiatives to reduce both insecticide price and improve efficiency of delivery systems [48].

The present work is amongst the few studies that have evaluated the cost and cost-effectiveness of combining malaria control interventions within a resource-constrained setting, particularly in a setting where emergent resistance to pyrethroids has reduced the programmatic effectiveness of standard, pyrethroid only ITNs. Thus, comparisons with other published works require caution. Based on other peer-reviewed studies published from 2000–2010, White et al., estimate a significantly lower ICER per DALY averted for bed nets (US$27, range US$8.15-US$110) and IRS (US$143, range US$135-US$150), but no combined ICER was reported due to lack of evidence [9]. A recent clinical trial in Ethiopia, which followed a similar methodological approach as this study, concluded that ITNs (ICER = US$207) dominated (were more cost-effective) than IRS, as well as their combined effect (ICER = US$1,403) in a low malaria transmission setting [10]. In Tanzania the estimation of a nation-wide implementation of IRS on top of nets reached an ICER of US$152.36 per malaria case averted compared to nets alone [11], which was not dissimilar to our ICER per DALY averted in children U5 when adding the community-wide protective effect of IRS. This evidence should be considered by donors, international organizations and national governments, along with entomological insecticide resistance data to inform a more efficient allocation of resources of current malaria control strategies.

This study utilizes rigorous prospective data to determine the parameter values and to conduct extensive sensitivity analyses. However, it is not exempt of important limitations. First, the study did not include complications related to malaria, such as anaemia or cerebral malaria, which would have affected the ICERs and cost-effectiveness outcomes. Second, a recent evaluation showed no added benefit of employing IRS with pirimiphos-methyl on top of ITNs with piperonyl butoxide [13]. Similarly, Kafy et al., showed that malaria incidence was reduced when adding IRS using carbamates but not pyrethroids on top of standard ITNs in an area with pyrethroid-resistant vectors [49]. The cost-effectiveness of IRS in relation to newer ITN products is an important area for future investigation. Third, this study used the standard GDP per capita thresholds to determine cost-effectiveness. However, more conservative approaches incorporating health opportunity costs would result in a maximum threshold of US$155 in Mozambique [50]. Under these thresholds, the adoption of IRS with pirimiphos-methyl would require a sustained and substantial price reduction beyond the lifespan of the NgenIRS partnership.

## Conclusions

This study provides evidence on the cost-effectiveness of adopting IRS with an effective insecticide, pirimiphos-methyl, in high-burden regions with high access to standard ITNs and pyrethroid resistance. Consistent with findings from other studies, this study illustrates the importance of delivering effective insecticides at high coverage for effective malaria control. However, the adoption of such a strategy in these settings should be accompanied by efforts to maintain reductions in the insecticide price, which is the main driver of IRS costs. These findings provide important evidence to inform global and national policies on how to implement vector control interventions to accelerate progress towards meeting global malaria targets.

## Supplementary Information

**Additional file 1**: Main outputs for the 2016 and 2017 spraying campaigns in the district of Mopeia.

**Additional file 2**: Univariate sensitivity analysis: changes on incremental cost-effectiveness ratios (ICERs) due to changes in selected parameter.

**Additional file 3**: Cost-effectiveness planes.

**Additional file 4**: Review of indoor residual spraying campaigns.

## Data Availability

The datasets generated and/or analysed during the current study are available in the *Dipòsit Digital de la Universitat de Barcelona* repository, http://diposit.ub.edu/dspace/handle/2445/101776.

## Abbreviations

CDC: US Centers for Disease Control and Prevention
CISM: Centro de Investigação em Saúde de Manhiça
DALY: Disability-adjusted life-year
DDT: Dichlorodiphenyltrichloroethane
GDP: Gross domestic product,
ICER: Incremental cost-effectiveness ratio
IRS: Indoor residual spraying
ITN: Insecticide-treated net
PMI: US President’s Malaria Initiative
U5: Children under 5 years of age
US$: United States dollar
WHO: World Health Organization
