# Supplementary material for: Cost and cost-effectiveness of indoor residual spraying with pirimiphos-methyl in a high malaria transmission district of Mozambique with high access to standard insecticide-treated nets
Source: Malar J. 2021 Mar 10;20:143. doi: 10.1186/s12936-021-03687-1 (PMC7948350; doi:10.1186/s12936-021-03687-1)
Supplement: Supplementary file 1 — Additional file 1: Main outputs for the 2016 and 2017 spraying campaigns in the district of Mopeia. [file 12936_2021_3687_MOESM1_ESM.docx]

**Additional file 1. Main outputs for the 2016 and 2017 spraying campaigns in the district of Mopeia**

|  | **2016 campaign** | **2017 campaign** |
| --- | --- | --- |
| Campaign operational days | 42 | 50 |
| Structures found by SOPs | 19,992 | 19,950 |
| Structures sprayed in Mopeia | 16,500 | 16,936 |
| Structures sprayed in Zambézia | 405,597 | 381,463 |
| Spray coverage of structures (Mopeia) | 82.53% | 84.89% |
| Total population found | 89,258 | 83,452 |
| Population protected | 76,669 | 70,988 |
| Males protected | 39,148 | 35,456 |
| Females protected | 37,521 | 35,532 |
| Pregnant women protected | 3,908 | 2,759 |
| Children under five years of age protected | 11,513 | 10,738 |
| % population protected | 85.90% | 85.06% |
| Insecticide bottles used | 10,603 | 14,633 |
