# Supplementary material for: Cost and cost-effectiveness of indoor residual spraying with pirimiphos-methyl in a high malaria transmission district of Mozambique with high access to standard insecticide-treated nets
Source: Malar J. 2021 Mar 10;20:143. doi: 10.1186/s12936-021-03687-1 (PMC7948350; doi:10.1186/s12936-021-03687-1)
Supplement: Supplementary file 2 — Additional file 2: Univariate sensitivity analysis: changes on incremental cost-effectiveness ratios (ICERs) due to changes in selected parameter. [file 12936_2021_3687_MOESM2_ESM.docx]

**Additional file 2. Univariate sensitivity analysis: changes on incremental cost-effectiveness ratios (ICERs) due to changes in selected parameter.** US$ (% change)

Results from the deterministic analysis using the most likely values (table 1). Cost-effective (CE) values expressed in green and highly cost-effective (HCE) in bold green. Threshold based on the (three times) Mozambican gross domestic product per capita in 2018. IRR=incidence rate ratio. IRS=indoor residual spraying.

| **Individual of all ages** | | | | **Children under five years of age (U5)** | | | |  |  |  |
| --- | --- | --- | --- | --- | --- | --- | --- | --- | --- | --- |
| **ICER - direct costs only** | | **ICER - direct and indirect costs** | | **ICER - direct costs only** | | **ICER - direct and indirect costs** | |  |  |  |
| 2,525 | Not CE | 1,822 | Not CE | 584 | CE | 404 | HCE | **Deterministic result of main analysis** | | |
| 3,957 | 57% | 3,254 | 79% | 657 | 12% | 509 | 26% | **Malaria incidence IRS (all ages)** | | 0.36696 |
| 1,832 | -27% | 1,128 | -38% | 519 | -11% | **315** | -22% |  |  | 0.30024 |
| -- | -- | -- | -- | 1,311 | 125% | 990 | 145% | **Malaria incidence IRS (children U5)** | | IRR=0.82 |
| -- | -- | -- | -- | **326** | -44% | **195** | -52% |  |  | 0.57708 |
| 2,933 | 16% | 2,230 | 22% | 908 | 55% | 690 | 71% | **Malaria prevalence (odd ratios)** | | all=0.70  childrenU5=0.54 |
| 3,870 | 53% | 3,166 | 74% | 902 | 54% | 722 | 79% | **IRS price** | | 12.39 |
| 1,702 | -33% | 998 | -45% | **389** | -33% | **209** | -48% |  |  | 7.06 |
| 3,526 | 40% | 2,542 | 40% | 630 | 8% | **435** | 8% | **Malaria mortality rate (all ages)** | | 0.0006134 |
| 2,306 | -9% | 1,664 | -9% | 559 | -4% | **387** | -4% |  |  | 0.0009603 |
| -- | -- | -- | -- | 690 | 18% | **476** | 18% | **Malaria mortality rate (children U5)** | | 0.004164 |
| -- | -- | -- | -- | 505 | -14% | **350** | -13% |  |  | 0.006246 |
| 3,004 | 19% | 2,169 | 19% | 580 | -1% | **412** | 2% | **Discount rate** | | 0% |
| 2,325 | -8% | 1,677 | -8% | 600 | 3% | **407** | 1% |  |  | 5% |
| 2,550 | 1% | 1,871 | 3% | 592 | 1% | **419** | 4% | **Health system costs (uncomplicated)** | | 3.54 |
| 2,501 | -1% | 1,774 | -3% | 577 | -1% | **389** | -4% |  |  | 5.12 |
| 2,527 | 0% | 1,829 | 0% | 584 | 0% | **406** | 0% | **% of severe cases** | | 0.004138561 |
| 2,514 | 0% | 1,773 | -3% | 581 | 0% | **392** | -3% |  |  | 0.012231257 |
