# Supplementary material for: Cost and cost-effectiveness of indoor residual spraying with pirimiphos-methyl in a high malaria transmission district of Mozambique with high access to standard insecticide-treated nets
Source: Malar J. 2021 Mar 10;20:143. doi: 10.1186/s12936-021-03687-1 (PMC7948350; doi:10.1186/s12936-021-03687-1)
Supplement: Supplementary file 3 — Additional file 3: Cost-effectiveness planes. [file 12936_2021_3687_MOESM3_ESM.docx]

| **Additional file 3. Cost-effectiveness planes.**  Results from the Monte Carlos simulations (10,000 iterations) obtained for the children U5 cohort (figures A and B) and the all age cohort (figures C and D). For the children U5 cohort 88% of the simulations laid below the highly cost-effectiveness threshold (US$ US$490.2) when considering indirect household costs (A) and 13% when disregarding them (B). In both cases 100% of the simulations lay below the cost-effectiveness threshold (US$1,470.60). For the all age cohort the number of iterations laying below the cost-effectiveness threshold reduce to 16% (C) and 0.02% (D) of them, using the IRS cost per person protected of 8.26 (range 7.25-9.27) obtained in the district.  CE=cost-effectiveness. Children U5=children under five years of age. DALY=disability-adjusted life-years. IRS=indoor-residual spraying. ITN=insecticide-treated net.  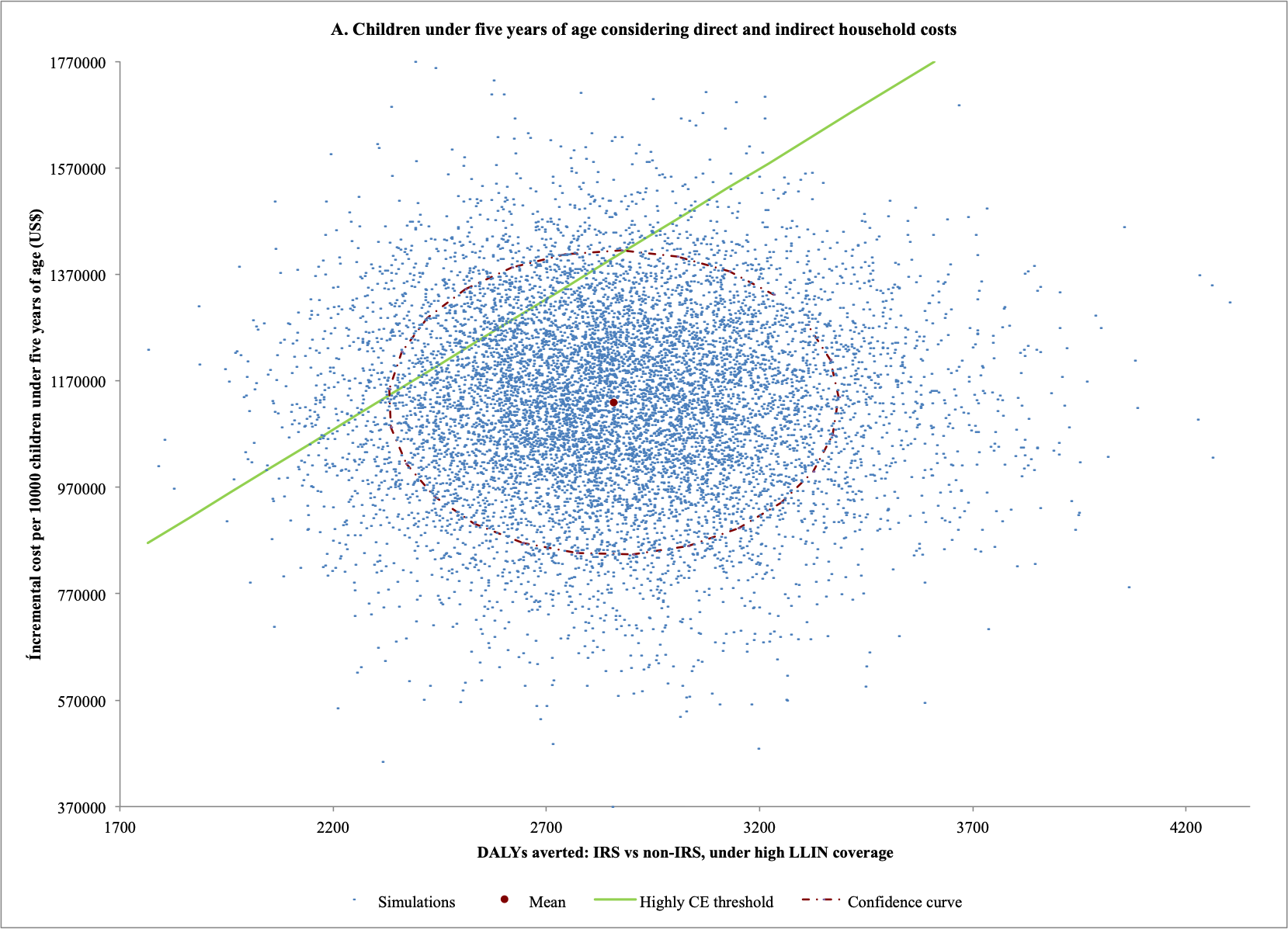 |
| --- |
| 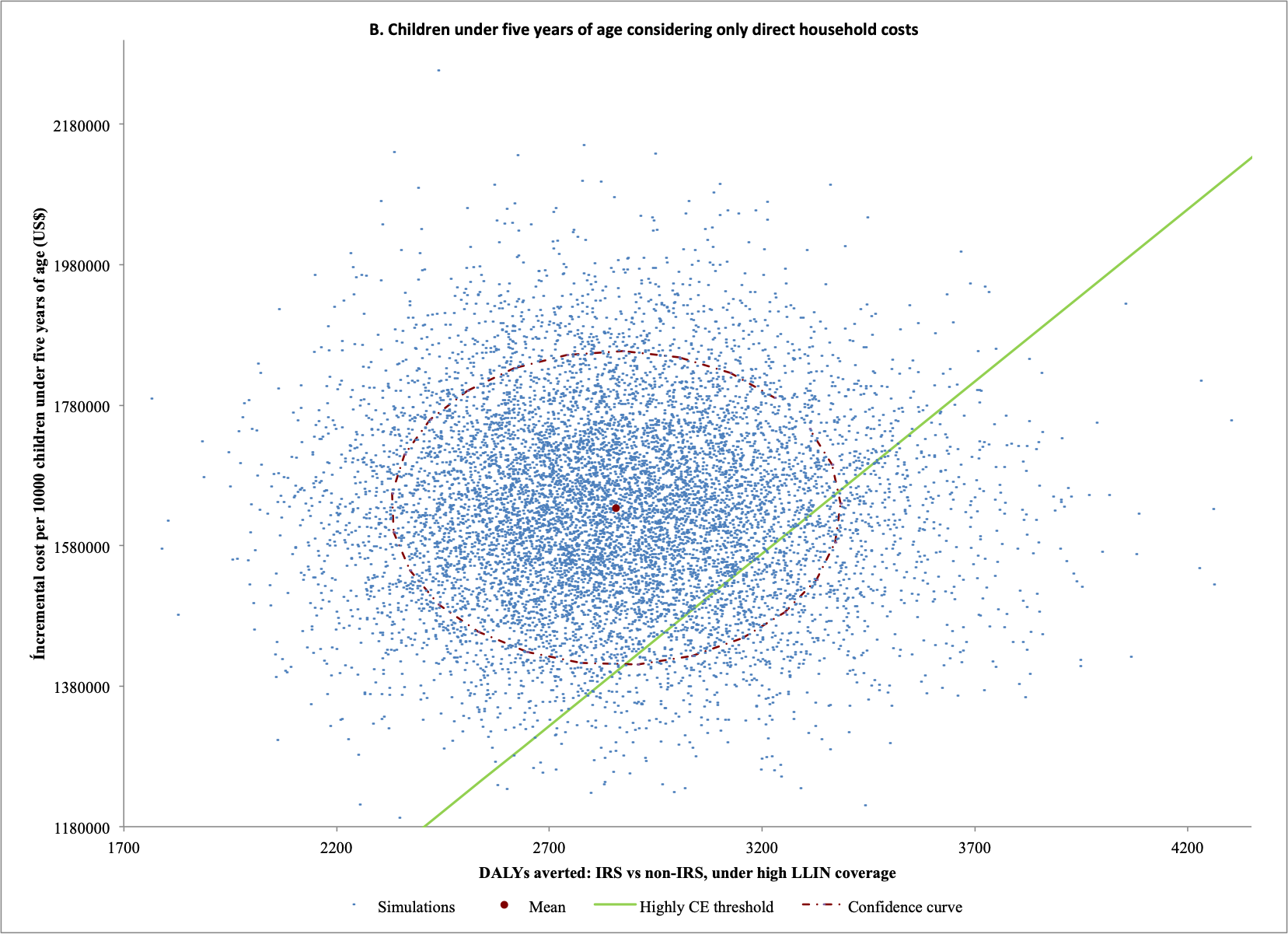 |
| 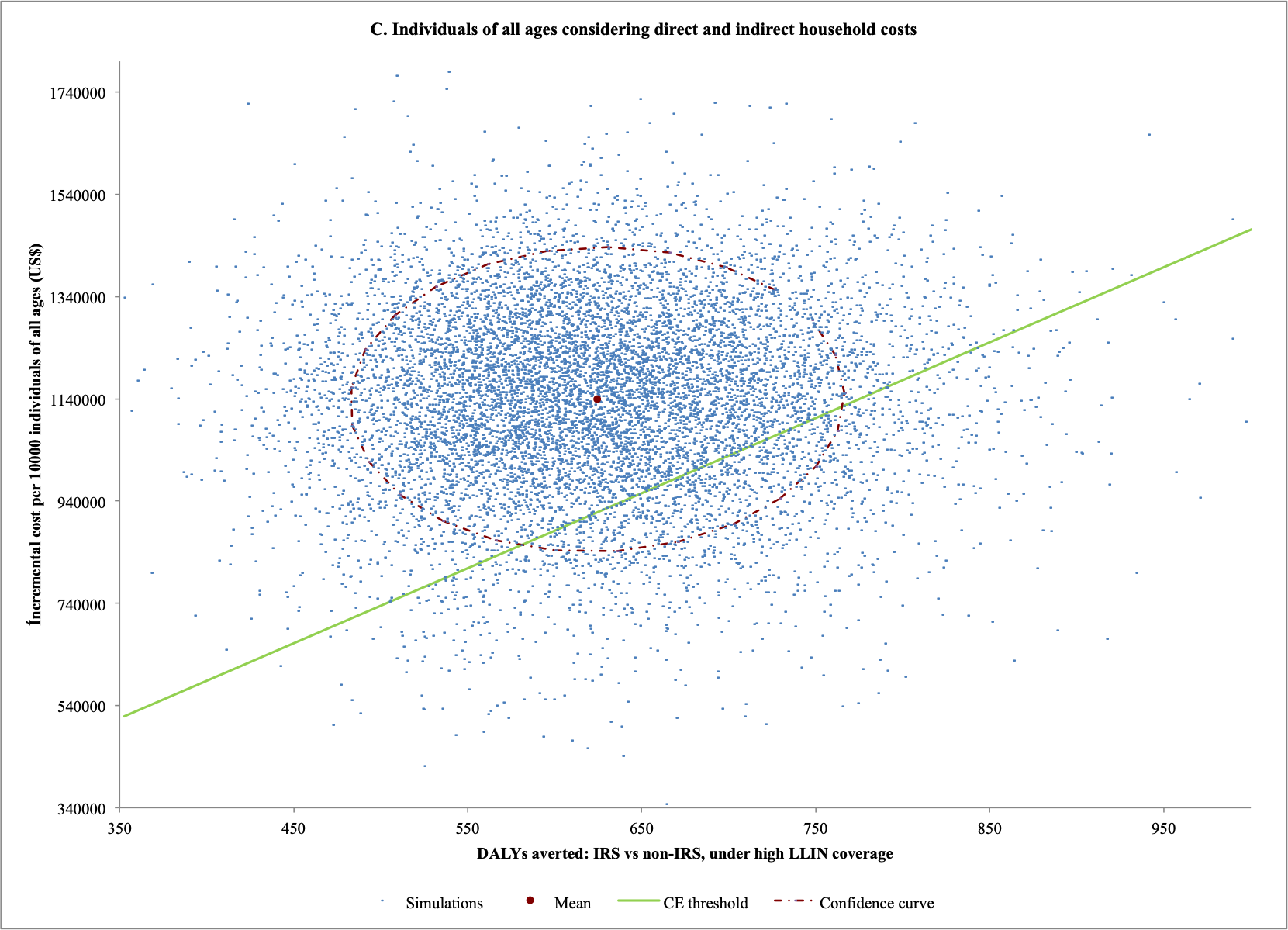 |
| 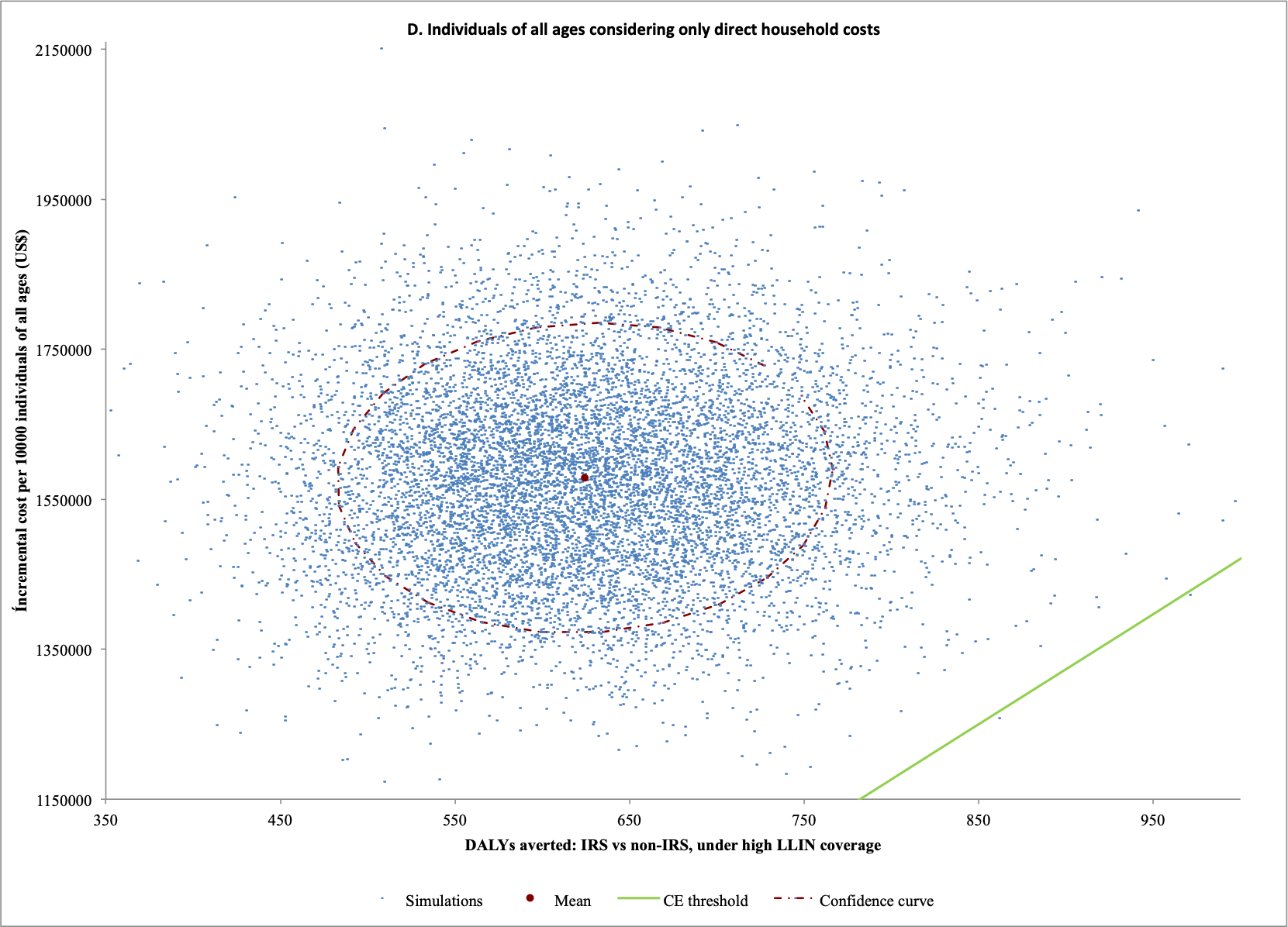 |
|  |
