# Supplementary material for: Cost and cost-effectiveness of indoor residual spraying with pirimiphos-methyl in a high malaria transmission district of Mozambique with high access to standard insecticide-treated nets
Source: Malar J. 2021 Mar 10;20:143. doi: 10.1186/s12936-021-03687-1 (PMC7948350; doi:10.1186/s12936-021-03687-1)
Supplement: Supplementary file 4 — Additional file 4: Review of indoor residual spraying campaigns. [file 12936_2021_3687_MOESM4_ESM.docx]

**Additional file 4. Review of indoor residual spraying campaigns (non-adjusted US$).** IRS=indoor residual spraying

|  | Cost/person protected | Insecticide | Year IRS | Country | First author | Ref |
| --- | --- | --- | --- | --- | --- | --- |
| **Our study** | **8.26** | **pirimiphos-methyl (organophosphate)** | **2016-7** | **Mozambique** | **Alonso S** | **--** |
| Literature Review | 6.70  (2.22-12.85) | Various | Various | Various | White MT | (1) |
| 1 | 3.07 | carbamate | 2014 | Ethiopia | Hailu A | (2) |
| 2 | 3.91 | DDT | 1994 | Thailand | Kamolratanakul P | (3) |
| 3 | 1.41 | pyrethroids | 1997 | India | Bhatia M | (4) |
| 4 | 4.82 | pyrethroids | 1999 | South Africa | Goodman CA | (5) |
| 5 | 9.25 | pyrethroids | 2001 | Colombia | Kroeger A | (6) |
| 6 | 12.87 | pyrethroids | 2001 | Colombia | Kroeger A | (6) |
| 7 | 1.54 | pyrethroids | 2000 | Zimbabwe | Worrall E | (7) |
| 8 | 1.74 | pyrethroids | 2000 | Kenya | Guyatt HL | (8) |
| 9 | 10.07 | carbamate | 2001 | Mozambique | Conteh L | (9) |
| 10 | 5.96 | carbamate | 2001 | Mozambique | Conteh L | (9) |
| 11 | 3.27 | DDT and pyrethroids | 1999 | South Africa | Yukich JO | (10) |
| 12 | 3.90 | DDT and carbamate | 2001 | Mozambique | Yukich JO | (10) |
| 13 | 2.50 | pyrethroids | 1996 | Tanzania | Curtis CF | (11) |
|  | | | | | | |

References:

(1) White MT, Conteh L, Cibulskis R, Ghani AC. Costs and cost-effectiveness of malaria control interventions - a systematic review. Malaria Journal. 2011;10(1):337. Available from: <http://malariajournal.biomedcentral.com/articles/10.1186/1475-2875-10-337>http://malariajournal.biomedcentral.com/articles/10.1186/1475-2875-10-337.

(2) Hailu A, Lindtjørn B, Deressa W, Gari T, Loha E, Robberstad B. Cost-effectiveness of a combined intervention of long lasting insecticidal nets and indoor residual spraying compared with each intervention alone for malaria prevention in Ethiopia. Cost Eff Resour Alloc.

(3) Kamolratanakul P, Butraporn P, Prasittisuk M, Prasittisuk C, Indaratna K. Cost-effectiveness and sustainability of lambdacyhalothrin-treated mosquito nets in comparison to DDT spraying for malaria control in western Thailand. Am J Trop Med Hyg. 2001;65(4):279–84.

(4) Bhatia M, Fox-Rushby J, Mills A. Cost-effectiveness of malaria control interventions when malaria mortality is low: Insecticide-treated nets versus in-house residual spraying in India. Soc Sci Med. 2004;59(3):525–39.

(5) Goodman CA, Mnzava AE, Dlamini SS, Sharp BL, Mthembu DJ, Gumede JK. Comparison of the cost and cost-effectiveness of insecticide-treated bednets and residual house-spraying in KwaZulu-Natal, South Africa. Trop Med Int Heal. 2001;6. Available from: <http://dx.doi.org/10.1046/j.1365-3156.2001.00700.x>http://dx.doi.org/10.1046/j.1365-3156.2001.00700.x

(6) Kroeger A, Ayala C, Medina Lara A. Unit costs for house spraying and bednet impregnation with residual insecticides in Colombia: a management tool for the control of vector-borne disease. Ann Trop Med Parasitol. 2002;96(4):405–16.

(7) Worrall E, Connor SJ, Thomson MC. Improving the cost-effectiveness of IRS with climate informed health surveillance systems. Malaria Journal. 2008;7:1–10.

(8) Guyatt HL, Kinnear J, Burini M, Snow RW. A comparative cost analysis of insecticide-treated nets and indoor residual spraying in highland Kenya. Health Policy and Planning. 2002;17. Available from: <http://dx.doi.org/10.1093/heapol/17.2.144>

(9) Conteh L, Sharp BL, Streat E, Barreto A, Konar S. The cost and cost-effectiveness of malaria vector control by residual insecticide house-spraying in southern Mozambique: a rural and urban analysis. Trop Med Int Heal. 2004;9. Available from: <http://dx.doi.org/10.1046/j.1365-3156.2003.01150.x>http://dx.doi.org/10.1046/j.1365-3156.2003.01150.x

(10) Yukich JO, Lengeler C, Tediosi F, Brown N, Mulligan J-A, Chavasse D, et al. Costs and consequences of large-scale vector control for malaria. Malaria Journal. 2008;7(1):258. Available from: <http://malariajournal.biomedcentral.com/articles/10.1186/1475-2875-7-258>http://malariajournal.biomedcentral.com/articles/10.1186/1475-2875-7-258.

(11) Curtis CF, Maxwell CA, Finch RJ, Njunwa KJ. A comparison of use of a pyrethroid either for house spraying or for bednet treatment against malaria vectors. Trop Med Int Heal. 1998;3. Available from: <http://dx.doi.org/10.1046/j.1365-3156.1998.00281.x>http://dx.doi.org/10.1046/j.1365-3156.1998.00281.x
